# Title: Misdiagnosis in young females – Autism versus Borderline Personality Disorders

**DOI:** 10.1192/j.eurpsy.2024.282

**Published:** 2024-08-27

**Authors:** H. Sholinghur

**Affiliations:** Psychiatry, Equilibrium Healthcare, Eleanor Hospital, Manchester, United Kingdom

## Abstract

**Introduction:**

The diagnostic overlap between Autism Spectrum Disorder (ASD) and Emotionally Unstable Personality Disorder (EUPD), commonly referred to as Borderline Personality Disorder (BPD), presents a considerable challenge in psychiatric practice, particularly for young females. These complexities are amplified by gender biases in the healthcare system and can lead to misdiagnosis, affecting both treatment planning and long-term outcomes.

**Objectives:**

There are differences and similarities between ASD and EUPD/BPD which encompass its own challenges in diagnosis and treatment

The presentation seeks to:

1. Offer an in-depth overview of the relationship between ASD and EUPD/BPD in young females.
2. Evaluate the diagnostic challenges associated with distinguishing between these two conditions.
3. Discuss the practical implications of misdiagnosis on treatment and quality of life.

**Methods:**

Drawing from a rich corpus of evidence, including longitudinal studies (e.g., Kerns et al., 2015; Gunderson et al., 2018), meta-analyses, and patient case studies, the presentation adopts a multidisciplinary approach. It utilises clinical interviews, validated diagnostic tools such as the Autism Diagnostic Observation Schedule (ADOS) and the Structured Clinical Interview for DSM-5 (SCID-5), as well as direct observation to provide a nuanced understanding of ASD and EUPD/BPD characteristics.

**Results:**

**Shared Characteristics:** Both ASD and EUPD/BPD manifest challenges in social functioning and mood regulation, supported by studies indicating sensory sensitivities and affective dysregulation in both conditions (Zanarini et al., 2019; Happé et al., 2019).

**Differentiating Factors:** ASD individuals often struggle with verbal and non-verbal communication, whereas those with EUPD/BPD may excel in these areas but display emotional volatility and unstable relationships, substantiated by differing neurobiological markers (King-Casas et al., 2008; Minshew & Williams, 2007).

**Misdiagnosis Risks:** The failure to correctly diagnose these conditions may lead to ineffective or potentially harmful treatment plans (Zanarini et al., 2013; Solomon et al., 2012).

**Necessity for Comprehensive Assessment:** A multimodal and culturally sensitive diagnostic approach is essential for accurate clinical evaluation (Mandy et al., 2012; Betancur et al., 2009).

**Conclusions:**

The complexities surrounding the accurate diagnosis of ASD and EUPD/BPD in young females necessitate a thorough and multifaceted approach. An incorrect diagnosis could have long-lasting implications, affecting not just the efficacy of therapeutic interventions but also the overall well-being and quality of life of the individual. This presentation underscores the critical importance of drawing from a robust body of evidence and utilising comprehensive diagnostic approaches to differentiate these conditions.

**Disclosure of Interest:**

None Declared

